# Toxicology and Biodegradability of a Phthalate-Free and Bio-Based Novel Plasticizer

**DOI:** 10.1155/2021/9970896

**Published:** 2021-07-12

**Authors:** S. Simar-Mentières, F. Nesslany, M.-L. Sola, S. Mortier, J.-M. Raimbault, F. Gondelle, L. Chabot, P. Pandard, D. Wils, A. Chentouf

**Affiliations:** ^1^Institut Pasteur de Lille-Laboratoire de Toxicologie Génétique, 1 Rue Du Pr. Calmette, Lille Cedex 59019, France; ^2^ERBC, Chemin de Montifault, Baugy 18800, France; ^3^Ineris-Direction des Risques Chroniques, Pôle VIVA, Unité EXES–Parc Technologique ALATA. BP 2, Verneuil-en Halatte 60550, France; ^4^Roquette Frères, Toxicology & Safety Unit-Nutrition & Health R&D, 1, Rue de La Haute Loge, Lestrem 62136, France

## Abstract

Phthalate esters, mainly di-ethylhexylphthalate (DEHP), represent a class of chemicals primarily used as plasticizers for polyvinyl chloride in a wide range of domestic and industrial applications. These phthalate esters are low-toxicity environmental contaminants. To address these drawbacks, POLYSORB® ID 37, a blend of diesters obtained from esterification of isosorbide with plant-based fatty acids, was developed. The company can now offer PVC manufacturers a new product which competes with phthalates and other such chemicals. The market for plasticizers is very important, and ROQUETTE intends to provide a more sustainable and safer product. Isosorbide diester is bio-based (made from glucose and vegetable fatty acids). This plasticizer is registered in REACH regulation for high volumes (>1000 T/year). Risk assessment was obtained by conducting a wide range of biodegradability and toxicological protocols, using rodent models, according to established guidelines. Overall, all of the toxicological and biodegradability studies demonstrated that POLYSORB® ID 37 is nontoxic to mammalian life and is readily biodegradable.

## 1. Introduction

The phthalate esters, quantitatively dominated by di-ethylhexylphthalate (DEHP), represent a class of chemicals used mainly as plasticizers for polyvinyl chloride (PVC) in a wide range of domestic and industrial applications. Phthalate esters are environmental contaminants that display low toxicity. However, the effects of these compounds on reproductive system have been well characterized in animals, with gonadal toxicity observed after high dose exposure. Moreover, transgeneration studies have demonstrated that the reproductive system of young animals is particularly sensitive to certain phthalates. Martino-Andrade and Chahoud [1] reported that the phenotypic alterations observed in male offspring rats exposed during the perinatal period have remarkable similarities with common human reproductive disorders, including cryptorchidism, hypospadias, and low-sperm counts in the mature animals. Recent results also indicate that high phthalate doses can adversely affect adult and developing female rats. Thus, the question involving phthalates is whether the human exposure level is sufficient to adversely disrupt male and/or female reproductive system. Even if phthalate toxicity in humans is rare, the development of a safer alternative product in terms of toxicity is of course desirable. Moreover, the persistence of phthalates in the environment presents a long-term concern.

### 1.1. Partial Review of Toxicity Studies on Some Phthalate Compounds

The US Consumer Product Safety Commission [2] presented a review of use, exposure, and toxicity data to investigate the potential health effects of phthalate substitutes. Five chemicals chosen for review have been cited as already being used in children's articles: acetyl tri-n-butyl citrate (ATBC), di(2-ethylhexyl) adipate (DEHA), 1,2-cyclohexanedicarboxylic acid, dinonyl ester (DINCH), trioctyl trimellitate (TOTM), and di(2-ethylhexyl) terephthalate (DEHT or DOTP).

Among the five chemicals presented in CPSC review, TOTM appears to have the lowest migration potential; however, no mobility data were available for DEHT. Additionally, probably because it is new to the plasticizer market, DINCH lacks extensive exposure and toxicology data but does appear to have low migration rates and poor solubility in water, earning it approval from several governments to be used as a food contact substance [2].

Overall, a significant amount of toxicity information is currently available on four of these five chemicals, although the quality of some studies is questionable. No published studies of DINCH were available [2]. These compounds and some of the relevant toxicity data are discussed in the following paragraphs.

Among the five chemicals, ATBC appears to be the least toxic as indicated by a relatively high NOAEL and lack of cancer effects. For ATBC, acute oral toxicity is very low, based on studies where no lethality was observed at doses up to 25,000 mg/kg bw in mice, 31,500 mg/kg bw in rats, and 52,500 mg/kg bw in cats [3, 4]. In guinea pigs, there was no evidence of toxicity after a single dermal exposure to 1250 mg/kg bw, but repeated application of 250 or 500 mg/kg bw/day was reported to affect body and liver weight [4, 5].

In chronic exposure studies performed in rats, NOAELs and LOAELs were highest for DEHA at 948 (*M*) and 1104 (F) mg/kg bw/day, and 1975 (*M*) and 2300 (F) mg/kg bw/day, respectively.

While a cancer bioassay in rats was negative, one in mice was positive, showing induction of liver tumors in both males and females [6]. It has been hypothesized that the observed mouse liver tumors are a result of peroxisome proliferation and, therefore, of uncertain relevance to humans [7–12]. Based on these considerations, IARC [10] concluded that DEHA was not classifiable as to its carcinogenicity in humans (Group 3). However, in a previous assessment verified in 1991, US EPA classified DEHA in weight-of-evidence (WOE) Group C as a possible human carcinogen and calculated an oral slope factor (OSF) of 1.2 × 10^−3^ mg/kg/day [13]. The acute toxicity of DEHA is low by oral, inhalation, or dermal exposure [6, 14]. The US EPA has set a Maximum Contaminant Level (MCL) for DEHA in drinking water at 3.4 × 10^−8^ per *μ*g/L and the oral reference dose (RfD) at 0.6 mg/kg/day [13].

Regarding ATBC, in repeated-dose studies, the NOAEL in rats was 1000 mg/kg/day and no LOAEL reached in the only chronic study available. There was no clear evidence of specific target organ toxicity. Possibly related to this observation are two studies which reported results suggestive of a nonadverse, adaptive response to ATBC in the liver (increased liver weight and/or hepatic hypertrophy) and possibly the kidney [15–17]. A 2-year dietary cancer bioassay in rats was negative, although perhaps not an adequate test of carcinogenicity because group sizes were relatively small (20 per treated group and 40 in controls); 20% of animals died early from infection, and doses were inadequate (the high dose did not approach the MTD) [18]. Thus, the usefulness of this 2-year study is limited due to the several deficiencies. Dietary reproductive toxicity tests in rats and mice did not reveal any effects of ATBC on reproductive parameters, such as fertility, mating, spermatogenesis, or gestation, or postnatal developmental effects [4, 17, 19].

Regarding DEHT, data are available on the subchronic, chronic, reproduction, and developmental toxicity of DEHT. For repeated-dose animal toxicity, there was no clear evidence of specific target organ toxicity of DEHT, although the subchronic, reproductive, and developmental studies in rats and mice reported results suggestive of a nonadverse, adaptive response to DEHT in the liver (increased liver weight) [20–22]. A 2-year dietary cancer bioassay in rats was negative [23]. In the reproductive toxicity studies, reductions in feed consumption and maternal and pup body weights were observed; however, no reproductive effects based on fertility, mating, estrous cycle lengths, gestation lengths, gender ratios, liver litter size, or postnatal survival were observed in rats during this study [20, 21]. DEHT had NOAELs from 324 to 102 mg/kg/d and LOAELs from 418 to 666 mg/kg/d for males and females, respectively.

No published studies of DINCH were found. The only information located regarding the health effects of DINCH was found in the SCENIHR [24] report, which contained summaries of unreferenced and unpublished studies submitted by BASF Corporation, and in an abstract/summary of one of these studies submitted by BASF Corporation to EPA under the Toxic Substances Control Act (TSCA) and identified in the search of the TSCATS database. Of these studies, the lowest LOAELs were reported by BASF to be 200 and 300 mg/kg/day for thyroid effects in the 2-year and 2-generation reproduction studies. Corresponding NOAELs were 40 and 100 mg/kg/day. The 2-generation study in rats showed no reproductive toxicity in either generation at doses as high as 1000 mg/kg/day, as reported by BASF.

Regarding TOTM, no subchronic or chronic study was available. [25–27]. The only study available found no effects on reproductive function but did report decreased spermatocyte and spermatid counts [27, 28]. TOTM did not induce developmental effects in rats following gavage treatment during gestation [29] and limited data in strain A mice suggest that TOTM is not a lung carcinogen [30]. However, this chemical is lacking rodent 2-year chronic toxicity studies.

In order to reduce human exposure to phthalates, the development of a POLYSORB® ID 37, a blend of diesters obtained from esterification of isosorbide with plant-based fatty acids, was performed. Thanks to its plasticizing properties equivalent to the phthalate products on the market, this product constitutes an alternative to the phthalates conventionally used for the manufacture of flexible PVC. In relation to the other phthalate-free plasticizers (adipates, acetylated monoglycerides, and citrates) it is particularly versatile and has both excellent compatibility with PVC and a very low volatility.

This article describes the toxicological and biodegradability studies carried out in order to realize the risk assessment for human and environment safety of this new plasticizer.

## 2. Materials and Methods

### 2.1. Test Article

POLYSORB® ID 37 (also named LAB 3822 in the current paper) is a blend of diesters obtained from esterification of isosorbide with plant-based fatty acids comprising caprylic acid (octanoic acid; C8) and capric acid (decanoic acid; C10) fatty acids.

The fatty acids come from an overhead fraction (C10/C8 mixture 35 to 45%/55 to 65) from vegetable oil. The part is used without adding product. They all come from plant products.

POLYSORB® ID 37 guarantees a good property on PVC and good accounting within several polymer matrices, being 100% bio-based from annually renewable raw materials.

**Figure 1:**
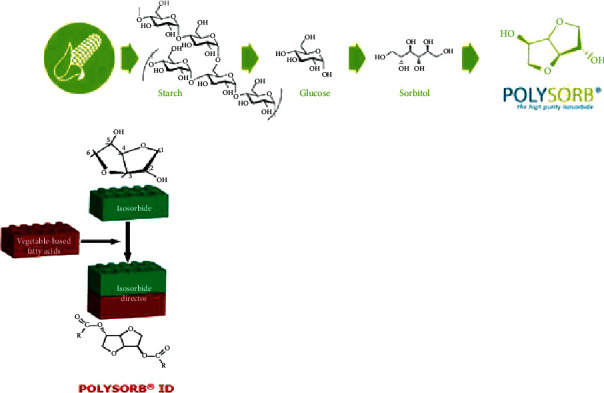
Synthesis of POLYSORB® ID 37 by esterification of isosorbide with plant- (corn-) based fatty acids.

Isosorbide is obtained from corn via three chemical processes, involving no harmful reactants (see Figure 1).

The characteristics and properties of POLYSORB® ID 37 are summarized in Table 1.

#### 2.1.1. Analytical Method Validation of POLYSORB® ID 37

POLYSORB® ID 37 comprises of a DEI (di-esters-isosorbide). After oral administration it has been hypothesized that the DEI is hydrolysed by pancreatic lipase to form isosorbide and free fatty acids; therefore the measurement of plasma isosorbide would be a marker of POLYSORB® ID 37 exposure. Gas chromatography-flame-ionization detection (GC-FID) was used to determine the level of isosorbide in rat plasma.

Briefly the analytical method used to validate the isosorbide assay included separation in split mode on a capillary column Varian DB1 (0.25 *μ*m) with the following temperature gradient: initial temperature: 120°C, ramp: 1°C/min to 128°C, 20°C/min to 268°C, 50°C/min to a final temperature of 300°C. The total flow was 300 mL/min and the split flow was 80 mL/min.

Blood samples from the rats were collected in lithium and heparin tubes, and the plasma was precipitated with trichloroacetic acid. At this step of the validation, isosorbide and the internal standard (methyl-alpha-D-glucopyranoside) were added. For the in vivo study samples, isosorbide was replaced with pyridine. The mixture was then evaporated and reconstituted in pyridine. The final extracts were derivatized with N,O-Bis(trimethylsilyl)trifluoroacetamide with 1% trimethylsilyl chloride, heated, and centrifugated. A sample size of 4 *μ*L from the final extracts was used for analysis in the GC-FID.

Results were obtained using STAR software (version 6.41) and expressed as the area ratio of the peak of isosorbide (retention time of 9.5 minutes) and the peak of internal standard (13.5 minutes).

### 2.2. Pharmacokinetic Parameters and Urinary Excretion of POLYSORB® ID 37 following a Single Oral Dose in Rats

This study was conducted to determine pharmacokinetic parameters and urinary excretion of isosorbide in the rat, following single oral administration of POLYSORB® ID 37.

After oral administration, the POLYSORB® ID 37 (i.e., DEI) is hydrolysed by the lipases, yielding isosorbide and free fatty acids.

Eight-week-old male and female Sprague-Dawley rats (Charles River Laboratories, Saint-Germain sur l'Arbresle, France), weighing 274.5–311.4 g for the males and 150.6–184.3 g for the females, were randomized in into either groups for plasma (4 groups of 12 males or females) or groups for urine (4 groups of 4 males or females).

Animals received by oral route 0.5, 1, or 2 g of DEI/kg body weight (bw) equivalent to 0.575 g, 1.15 g, or 2.3 g of POLYSORB® ID 37/kg bw, respectively. One group orally received isosorbide 2 g/kg bw. Mortality and morbidity were recorded twice a day. General observations were performed before the first dosing and at 60 minutes after dose (±30 minutes) on day 1 (D1) and once daily to the end of the study. Pharmacokinetic blood samples were drawn on D1 (before dose and at 0.25, 0.5, 1, 2, 4, 8, 10, 24, 48, and 72 h after dose). Twenty-four hours' urine samples were collected for 24 hours prior to dosing and then at 24, 48, and 72 hours after dosing.

### 2.3. Toxicology Testing

All toxicology studies were performed according to the guidelines for Good Laboratory Practice (GLP) published by OECD (ENV/MC/CHEM (98)) [31] and ENV/JM/MONO (2002)9 [32] and published by French Ministry of Health (March 14^th^, 2000) and EC Commission Directive 2004/10/EC [33].

#### 2.3.1. Acute Toxicity

Qualitative and/or quantitative evaluation of toxic effects was done after a single intravenous administration of POLYSORB® ID 37 in both male and female mice and rats based on the general requirements of the OECD Guideline No. 423 [34].

Five groups of 10 (5 male and 5 female), 7-8-week-old SPF Swiss mice or SPF Sprague-Dawley rats (Charles River Laboratories, Saint-Germain sur l'Arbresle, France) were randomized and weighed (mice: 32.0–39.3 g male, 22.9–29.0 g females; rats: 271.8–310.9 g males, 210.2–21.8 g females). Doses of 0.25, 0.5, 1, or 2 g DEI/kg bw (0.29, 0.575, 1.15, or 2.3 mg POLYSORB® ID 37/kg bw) were either administered orally or intravenously without dilution in sterile water. The control group received 0.9% sodium chloride solution.

#### 2.3.2. Local Tolerance: Murine Local Lymph Node Assay

The skin sensitising potential of POLYSORB® ID 37 was evaluated in female mouse using an *in vivo* nonradioactive Local Lymph Node Assay (LLNA) in accordance with the General Requirements of OECD Guideline No. 429 [35].

Sixteen female CBA/J (Centre d'Elevage R. Janvier, Le Genest St Isle, France) mice were randomized to one of four groups: vehicle group (acetone/olive oil), undiluted POLYSORB® ID 37, acetone, the positive control vehicle, or positive control group receiving 0.5% (w/v) 2,4-dinitrochlorobenzene (DNCB) in acetone.

### 2.4. Repeated Toxicity Studies

#### 2.4.1. Twenty-Eight-Day Toxicity Study in the Rat

The study was conducted in accordance with OECD Guideline for Testing of Chemicals No. 407 [36].

Six- to 10-week-old, SPF Sprague-Dawley rats (Charles River Laboratories France, Saint-Germain sur l'Arbresle, France) were randomized into one of five groups, each consisting of 10 males and 10 females. Groups 1, 2, and 5 included 10 males and 10 females for withdrawal and Groups 1, 3, 4, and 5 included 6 males and 6 females as satellite groups. On the day of randomization, the males weighed between 250.3 and 298.9 g, and the females between 190.0 and 225.1 g. No vehicle was used for the study since POLYSORB® ID 37 was supplied formulated. Sterile water (Baxter, Guyancourt, France) was used for the negative control group. A mixture of capric (C8) and caprylic acid (C10) was used in the reference group. Water, the mixture of fatty acids, and POLYSORB® ID 37 were administered by oral gavage for 28 days.

The control group (Group 1) received 2.35 mL sterile water/kg bw/day. Group 2 received 1.4 g of C8/C10/kg bw/day while Groups 3–5 were treated with 0.5, 1, or 2 g DEI/kg bw/day, respectively. Animals were treated daily, at approximately the same time daily, with a volume of 2.35, 1.57, 0.59, 1.17, and 2.35 mL/kg bw for Groups 1, 2, 3, 4, and 5, respectively. The doses were selected based on previous studies (results not presented). Reference item (C8/C10) dose represents about 2/3 of the highest volume administered to the treated animals (i.e., 2.35 mL/kg bw); according to the proportion of C8/C10 in the POLYSORB® ID 37.4 satellite additional groups (each with 6 males and 6 females) were dosed with the vehicle, the lowest, intermediate, and highest dose of POLYSORB® ID 37. These animals were used for toxicokinetic assessment only (no other observation was performed). Since effects were assessed during the last week of treatment, animals from the withdrawal group were observed using the same battery during the second week of the withdrawal period (i.e., D42).

Ophthalmological examination (retinography) was performed on all animals from the main group before the first dose and D28, and before the first dose, D28, and D42 in the withdrawal.

The day prior to the blood sample collection, animals were fasted, and urine samples were collected from each animal. Blood samples were collected on D29 in the main groups and on D29 and D43 in the withdrawal groups. A complete set of hematology, coagulation, and clinical chemistry parameters was determined.

Tissue samples were collected at necroscopy from all animals for gross pathological and histopathological examination and evaluation. Selected organs, tissues, and any observed lesions from the control and highest dose level groups (10 rats/group) were forwarded for histopathological evaluation and reporting by a board-certified pathologist.

Isosorbide blood analysis was conducted on sample from satellite animals, with 3 males and 3 females sampled at each time point/group. Blood samples were drawn from the retroorbital sinus under isoflurane gas anaesthesia. The blood samples were cooled on ice and plasma was prepared within 60 minutes of sampling. The identity and concentration of the test article in the samples and the absence of the test article in the control samples were determined by GC-FID.

#### 2.4.2. Thirteen-Week Toxicity Study in the Rat

The study was conducted in accordance with OECD Guideline for Testing of Chemicals No. 408 [37].

This study followed the same protocol as for the 28-day study described above with the following exceptions: the C8/C10 group was eliminated; 15 males and 15 females were treated with the same DEI dose levels of 0, 0.5, 1, and 2 g/kg bw/day. A study on spermatogenesis was performed at the end of the treatment period. On the day of necropsy, sperm mobility, count, morphology, and viability of spermatozoa were assessed in all males in the main study only.

Concerning toxicokinetic analysis, blood samples were taken on D1 (before dose and at 30 min, 1 h, 2 h, 4 h, and 8 h after dose), on D28 before dose, and on D91 (before dose and at 30 min, 1 h, 2 h, 4 h, and 8 h after dose).

### 2.5. Embryo-Foetal Toxicity Study in the Rat

Adverse effects or disturbance on the embryo-foetal development observed during the repeated oral administration of the test item during pregnancy are evaluated in the female rat in accordance with general recommendations found in OECD Guideline No. 414 [38] adopted on January 22, 2001, and in the International Conference on Harmonisation of Technical Requirements for Registration of Pharmaceuticals for Human Use (ICH) and especially in the notes S5A and S5B [39]. Four groups (*n* = 24–25) of 10-11-week-old, mated SPF Sprague-Dawley female rats (weighing 214.2–296.3 g, Charles River, Saint-Germain sur l'Arbresle, France) and 4 satellite groups of 4 mated female rats were randomized to one of 5 treatments.

Four groups were dosed with either the vehicle, i.e., 0 g/kg (2.35 mL/kg bw. sterile water), the lowest dose of 0.5 g DEI/kg bw (0.575 g of POLYSORB® ID 37/kg bw or 0.59 mL/kg bw), an intermediate dose of 1 g DEI/kg bw (1.15 g of POLYSORB® ID 37/kg bw or 1.17 mL/kg bw), or the highest dose of 2 g DEI/kg bw (2.3 of POLYSORB® ID 37 g/kg or 2.35 mL/kg bw).

In parallel in the satellite groups, 4 mated females/group received the same treatment and were used for toxicokinetic assessment only. POLYSORB® ID 37 or sterile water was administered once a day at each chosen dose level, by the oral route from D6 to D19 of pregnancy (*i.e.*, from implantation to one day before termination).

Blood samples for isosorbide analysis were taken from satellite animals. Four females were sampled per time point and per group on D6 and on D19 (before dosing and then at 2 and 4 hours after dosing).

### 2.6. Genetic Toxicology

#### 2.6.1. Bacterial Reverse Mutation Assay (Ames Test)

POLYSORB® ID 37 was assayed via the Ames test according to OECD Guideline for Testing of Chemicals No. 471 [40] for gene mutation capability in five histidine-requiring strains of *Salmonella typhimurium* (TA98, TA100, TA1535, TA1537, and TA102; Dr. Bruce Ames). The Aroclor 1254 induced rat liver postmitochondrial fraction was obtained from Institut Pasteur de Lille, Lille, France. All treatments were performed with POLYSORB® ID 37 formulated in dimethylsulfoxide (DMSO). Test concentrations for the dose range-finding experiment were 50, 150, 500, 1500, and 5000 µg/plate (expressed as POLYSORB® ID 37) and 50, 150, 500, 1500, and 3000 or 5000 µg/plate (depending on the strain and the metabolic activation condition).

#### 2.6.2. Mouse Lymphoma Thymidine Kinase Gene Mutation Assay

POLYSORB® ID 37 was assayed in the mouse lymphoma TK gene mutation assay according to OECD Guidelines for Testing of Chemicals No. 476 [41]. Mouse Lymphoma L5178Y cells were obtained from the American Type Culture Collection (Sophia-Antipolis, France). The POLYSORB® ID 37 was solubilized in DMSO (Merck, Darmstadt, Germany), and the negative control consisted of DMSO alone. The S9 liver microsomal fraction used for metabolic activation was prepared at Institut Pasteur de Lille (Lille, France).

In a preliminary toxicity study, in the absence of metabolic activation, POLYSORB® ID 37 was assayed using a 3 h exposure period at concentrations of 296.3, 444.4, 666.7, 1000, and 1500 *μ*g/mL. In Experiments 1 and 2, with metabolic activation, the POLYSORB® ID 37 was assayed at concentrations of 493.3, 740.7, 111.1, 1666.7, and 2500 *μ*g/mL for a 3 h exposure period. During testing without metabolic activation, i.e., Experiment 2, the exposure period was extended to 24 h, using concentrations of 125, 187.5, 250, 375, 70, 82.5, 92.5, and 500 *μ*g/mL.

#### 2.6.3. Mammalian Erythrocyte Bone Marrow Micronucleus Test

The study followed the guideline set forth by OECD Guideline for Testing of Chemicals No. 474 [42].

Male and female Sprague-Dawley rats (159 g–192 g (males) and 129 g to 157 g (females); Charles River Laboratories, Saint-Germain sur l'Arbresle, France) were randomized to POLYSORB® ID 37 doses of 500, 1000, or 2000 mg/kg bw. The animals were orally gavaged with POLYSORB® ID 37 at 24 and 48 hours before sampling. Plasma levels of isosorbide were measured after exposure at 30 minutes or 2 or 4 hours using a validated analytical method in animal satellite groups.

### 2.7. Ready Biodegradability CO_2_ Evolution (Modified Sturm Test)

The tests were performed according to OECD Guideline for Testing of Chemicals No. 301 [43].

The POLYSORB® ID 37 has been incorporated directly in the mineral medium to get an initial nominal test concentration of 10 and 20 mg/L (OC) by weighing an adequate quantity.

The inoculum for testing was prepared from activated sludge sampled in the aeration tank treatment plant receiving predominantly domestic sewage. A measured volume of inoculated mineral medium containing a known concentration of POLYSORB® ID 37 or isosorbide as the nominal sole source of organic carbon was aerated by the passage of carbon dioxide-free air at a controlled rate in the dark. Degradation was followed over the 28-day incubation time by determining the carbon dioxide produced.

The carbon dioxide (CO_2_) released from mineralization is trapped as BaCO_3_ in barium hydroxide (Ba (OH)_2_) and is measured by titration of the residual 0.0125 M Ba (OH)_2_ with a 0.05 M HCl solution in presence of phenolphthalein.

The amount of CO_2_ produced from test article is corrected for that derived from blank control and then expressed as a percentage of the theoretical CO_2_ (%Th CO_2_).

## 3. Results

### 3.1. Chemical Analyses of POLYSORB® ID 37

The validation of the method to determine isosorbide in plasma and urine of rats showed the following.

  Good selectivity of the assay: there was no interaction at the retention time of isosorbide.
  Good calibration range between the concentrations around 10.8 and 215.6 *μ*g/mL in plasma and around 100 and 8000 *μ*g/mL in urine (regression coefficient > 0.990 and back calculation in the range 85–115% for level different from limit of quantification (LOQ) and 80–120% for the LOQ).
  Good intra- and interassay precision (≤20%) for quality control (QC) of 10 *μ*g/mL (LOQ) in plasma and for QC at 120 *μ*g/mL in urine, within the range of 80–120%.
  Good intra- and interassay precision (≤15%) for QC between 67.1 and 201.2 *μ*g/mL in plasma and *L* between 1000 and 8000 *μ*g/mL in urine, within the range of 85–115%.
  Good stability up to 4 months at −20°C, with good recovery within the range 85–115% of the concentration for level different from LOQ and 80–120% for LOQ level.
  Good stability for 24 hours at room temperature in urine, with good recovery within the range 85–115% of the initial mean concentration for level different from LOQ and 80–120% for LOQ level.
  Good stability in urine for freeze and thaw and short-term assays, with good recovery within the range 85–115% of the initial mean concentration for level different from LOQ and 80–120% for LOQ level.
  The limit of quantification was defined at 10 *μ*g/mL in plasma and 120 *μ*g/mL in urine.
  The extraction yields were 73.0%.
  No dilution effect was detected.

The analytical method was validated within the range 85–115% of the theoretical concentration and with a precision lower than 15% for levels greater than LOQ and within the range 80–120% of the theoretical concentration and with a precision of less than 20% for the LOQ.

For the in vivo study samples, two QC2, two QC3, and two QC4 will be used and at least four of them will have to be within the range 85–115% of their theoretical concentration to accept the series.

### 3.2. Pharmacokinetic and Urinary Excretion Study in Rats following Single Oral Administration

Toxicokinetic (TK) analysis was conducted using the mean isosorbide concentrations from three males and three females per group and at each time point using a noncompartmental analysis (NCA). The linearity of exposure was evaluated from comparison of the AUC/Dose and *C*_max_/Dose ratios.

Concentrations of isosorbide were found to be lower than the limit of detection and lower than the limit of quantification in all predose samples. Toxicokinetic results confirmed that all treated animals in both genders were exposed to POLYSORB® ID 37 with a moderate interindividual variability. Peak plasma concentration *C*_max_ was observed between 0.5 and 2 hours after dosing for males and at 1 hour after dosing for females. The half-life of POLYSORB® ID 37 was between 1.9 and 3.4 hours.

Comparison of the AUC_all_ of POLYSORB® ID 37 or isosorbide in males and females (from 1.43 to 1.75) suggests that males were slightly more exposed to POLYSORB® ID 37 or isosorbide than females. Furthermore, AUC_last_ increased with the dose level. There was a good linearity between exposure or *C*_max_ and the dose levels.

The maximum observed urinary excretion rate and the AUC _all_ also increased with the dose of POLYSORB® ID 37. The amount of POLYSORB® ID 37 found in urine was greater in males compared to females regardless of dose. Compared to POLYSORB® ID 37 at 2 g of DEI/kg bw values of all urinary parameters of isosorbide administered at 2 g/kg bw were 2 times as high. In summary, animals dosed with POLYSORB® ID 37 (0.5, 1, and 2 g of DEI/kg bw) were exposed to POLYSORB® ID 37, and standard pharmacokinetic parameters were determined.

### 3.3. Toxicology Testing

#### 3.3.1. Acute Toxicity

POLYSORB® ID 37 (batch FAL 07/25) administered once by oral gavage in male and female Swiss mice and in the male and female Sprague-Dawley rats did not induce any mortality or sign of toxicity up to the dose of 2 g/kg bw of DEI (2.3 g/kg bw of POLYSORB® ID 37).

Likewise, intravenous administration of POLYSORB® ID 37 (batch FAL 07/25) in male and female Swiss mice did not induce any mortality. However, some signs of toxicity including absence of locomotor activity or of piloerection were observed on D1 at and above 0.5 g/kg bw of DEI (0.575 g/kg bw of POLYSORB® ID 37); therefore, the maximum nonlethal dose is 2 g/kg bw of DEI (2.3 g/kg bw of POLYSORB® ID 37).

POLYSORB® ID 37 (batch FAL 07/25) induced mortality in male Sprague-Dawley rats when administered once by the intravenous route at 1 g/kg bw and 2 g/kg bw DEI and in female Sprague-Dawley rats when administered once by the intravenous route at 2 g/kg bw DEI. Moreover, signs of toxicity began to appear in the male Sprague-Dawley rats dosed with POLYSORB® ID 37 at 0.5 g/kg bw DEI and in the female Sprague-Dawley rats dosed with POLYSORB® ID 37 at 1 g/kg bw DEI. As a result, the maximum nonlethal dose is 0.5 g/kg bw of DEI (0.575 g/kg bw of POLYSORB® ID 37) in male Sprague-Dawley rats and 1 g/kg bw DEI (1.15 g/kg bw of POLYSORB® ID 37) in female Sprague-Dawley rats by intravenous route.

#### 3.3.2. Local Tolerance: Murine Local Lymph Node Assay

On D3, a discrete erythema was noted on both ears of one animal and one ear of one animal treated with the test article POLYSORB® ID 37 100%. It did not produce a stimulation index equal to or greater than 3 at any of the doses tested. Consequently, the Estimated Concentration 3 (EC3) value for POLYSORB® ID 37 cannot be calculated. Under the experimental conditions adopted, POLYSORB® ID 37 did not induce delayed contact hypersensitivity in the murine local lymph node.

### 3.4. Repeat Dose Toxicity Studies

#### 3.4.1. Twenty-Eight-Day Toxicity Study in the Rat

In the 28-day repeated-dose oral toxicity rat study, mortalities (6 males and 6 females) occurred in Group 2 which was dosed with C8/C10. Due to difficulties in method of administration (i.e., use of a glass syringe) the treatment and administration method were modified. In the POLYSORB® ID 37 at 0.5 g DEI/kg bw, one male was found dead on D25 prior to dosing. No abnormalities were noted at the macroscopic examination.

Body weight gain was lower in males and females dosed with C8/C10 from D7 to D28 which was correlated to the lower tolerance of C8/C10. Body weight of male animals dosed with POLYSORB® ID 37, at all doses tested, had a tendency to increase compared to the negative control group.

Clinical signs were observed in group dosed with C8/C10. Bradypnea and reduced spontaneous locomotor activity were observed in male and female animals. Furthermore, signs of passivity were noted in females. These signs have also been observed in animals during the reversibility period at a lower frequency. During week 1, the animals in Group 2 (C8/C10) had decreased food and water consumption compared to control group.

In male animals, POLYSORB® ID 37, at all tested doses, led to an increase in triglycerides that reached significance at 2 g of DEI/kg bw. POLYSORB® ID 37, at all doses, showed a trend of a decrease of total bilirubin. At 2 g of DEI/kg bw, there was an increase in glucose concentration and a decrease in lipase activity. In female animals, triglyceride concentration and gamma glutamyl transferase activity were increased only in animals dosed with fatty acid C8/C10, while there was a tendency for a decrease in lipase activity noted in all dose groups, although it was less sustained after dosing with the top dose at 2 g of DEI/kg bw and in the reference group. Males dosed with POLYSORB® ID 37 also had increased triglycerides after dosing at 2 g of DEI/kg bw. Females in the 2 g DEI/kg bw POLYSORB® ID 37 group and those in the C8/C10 group had an increase in potassium on D29 and D43, respectively.

There were no changes in osmolality or in urinary parameters in animals dosed with POLYSORB® ID 37 at 0.5, 1, and 2 g equivalent DEI/kg bw. Males dosed with C8/C10 had a decrease in urinary volume on D29 (−25%) and D43 (−23%). Males dosed with POLYSORB® ID 37 at 0.5 g equivalent DEI/kg had an increase in urinary volume on D29 (+33%). No other changes were noted in urinary volume in females and in males.

There was no observed effect on body temperature in any of the groups (vehicle or POLYSORB® ID 37 at 0.5, 1, or 2 g of DEI/kg bw/day). Hematology and coagulation parameters were not affected by C8/C10 or with POLYSORB® ID 37 at 0.5, 1, or 2 g of DEI/kg bw/day.

Treatment with POLYSORB® ID 37 at 1 g of DEI/kg bw for 28 days resulted in a slight keratinized gastric hyperplasia in a single case, an incidence of little significance; however, POLYSORB® ID 37 at 2 g DEI/kg bw led to a slight keratinized gastric hyperplasia which completely recovered 14 days without treatment. Treatment with C8/C10 resulted in more severe keratinized gastric hyperplasia, ulceration of gastric and respiratory epithelia, and sometimes death; the gastric changes remained at a diminished degree after a 14-day period of recovery.

Toxicokinetic results confirmed that all treated animals were exposed to POLYSORB® ID 37 with a moderate interindividual variability. Comparison of exposure (AUC_last_) of POLYSORB® ID 37 in males and females suggested that there was no noticeable gender effect. The AUC_last_ increased with the dose level and were comparable between D1 and D28. The ratios of AUC in male and female rats suggest that there was no accumulation. There was a good linearity between exposure or *C*_max_ and the dose levels.

Taken together, C8/C10 administered at 1.4 g/kg bw led to mortality and modifications of clinical signs and histopathology (mainly gastric changes) and therefore was less tolerated than POLYSORB® ID 37. Despite a slight keratinized gastric hyperplasia at the doses of 1 and 2 g of DEI/kg bw, POLYSORB® ID 37 administered at the doses of 0.5, 1, and 2 g of DEI/kg bw did not induce any other sign of toxicity. Therefore, the No-Observed-Adverse Effect Level (NOAEL) of POLYSORB® ID 37 corresponds to 2 g of DEI/kg bw and the No-Observed-Effect Level (NOEL) to 1 g of DEI/kg bw.

#### 3.4.2. Thirteen-Week Toxicity Study in the Rat

In the 13-week repeated-dose oral toxicity study, only 3 males (1 in each dose group) and 3 females (1 in Group 1 and 2 in Group 4) died during the course of the study. Death was attributed to a gavage accident.

When compared to the control group, body weight gain was lower for males dosed with either 1 or 2 g of DEI/kg bw POLYSORB® ID 37 from D49 to D91 (between about −5% and 10%, when compared to the control group). In the withdrawal groups, body weight gain remained lower in the 1 and 2 g of DEI/kg bw POLYSORB® ID 37 groups and was associated with a slight decrease in food consumption. There was no effect on body weight and food and water consumption for the female rats in any of the groups.

No relevant toxicological clinical signs were observed whatever the dose of POLYSORB® ID 37, nor were there any changes in body temperature.

In male animals, POLYSORB® ID 37 administered at all doses had a trend to increase triglycerides, which was significant on D29 and D92 at the dose of 2 g of DEI/kg bw. In the females, triglycerides also increased at the dose of 2 g of DEI/kg bw on D29 only. No relevant toxicological changes in biochemical chemistry, hematology, coagulation parameters, or urinalysis were noted.

Toxicokinetic results confirmed that all treated animals were exposed to POLYSORB® ID 37 on D1 and D91. AUC_all_ of LAB 3822 or isosorbide in males and females (from 1.43 to 1.75) suggests that males were slightly more exposed to LAB 3822 or isosorbide than females. Furthermore, AUC_last_ increased with the dose level and there was good linearity between exposure or *C*_max_ and the dose levels.

No relevant abnormality was observed upon macroscopic examination and no relevant change in organ weight. There was a 30% *d* 28% decrease in both mobile spermatozoa and total number of spermatozoa, respectively, compared to the control in the 2 g of DEI/kg bw POLYSORB® ID 37; however there was no effect at either the 0.5 or 1 g of DEI/kg bw. A lower number in the tail of epididymis (but not in the testis) was noted at 1 g of DEI/kg bw when compared to the control group but not in the 2 other treated groups. Finally, statistical significance in the analysis of the morphology was noted in groups dosed with POLYSORB® ID 37 at 0.5 g of DEI/kg bw and at 1 g of DEI/kg bw. However, in absence of dose dependency, these latter changes were not considered as toxicologically relevant.

Under the experimental conditions, POLYSORB® ID 37 administered at the doses of 0.5, 1, and 2 g of DEI/kg bw for 13 weeks did not induce any sign of toxicity. Therefore, the NOAEL of POLYSORB® ID 37 corresponds to at least 2 g of DEI/kg bw.

### 3.5. Embryo-Foetal Toxicity Study in the Rat

Neither POLYSORB® ID 37 nor the vehicle was associated with mortality or clinical signs regardless of dose. Food consumption was slightly decreased in all animals exposed to POLYSORB® ID 37. This small decrease was not associated with a decrease in body weight gain and may be linked to the energy contribution from the fatty acids released by the LAB 3832. During macroscopic examinations, there were no observed organ abnormalities associated with either the sterile water or POLYSORB® ID 37 (0.5, 1, and 2 g DEI/kg bw). There was no effect on uterus weight compared to the control animals.

No differences were found in preimplantation loss, number of live foetuses, postimplantation loss, and foetal incidence in any of the POLYSORB® ID 37 (0.5, 1, and 2 DEI/kg bw) groups compared to the controls. At the time of caesarean delivery, no dead foetuses were found in either the control or the POLYSORB® ID 37 groups. No significant external abnormalities or any differences between foetal parameters were noted in the control and POLYSORB® ID 37 groups.

Examination of Bouin-fixed foetuses following freehand serial sectioning did not reveal any changes considered to be related to maternal treatment with POLYSORB® ID 37. Alizarin stained foetal skeletal preparations indicated a marginal reduction in ossification parameters at 2.0 g DEI kg bw/day which was a consequence of a slightly higher incidence of low body weight foetuses (<3.0 g) at this dose level. The incidence of low body weight foetuses was higher in Group 4 than in the other groups (in total 19 foetuses in Group 4 compared with 7, 3, and 12 foetuses in Groups 1, 2, and 3, respectively). For Groups 1–4, 4, 1, 4, and 10 respectively were skeletal preparations. The slight reduction in certain group mean ossification parameters in Group 4 likely resulted from a small number of low body weight foetuses which were not indicative of a generalised retardation of ossification in this group. With this exception, there were no skeletal changes that were considered to be related to maternal treatment with POLYSORB® ID 37.

Overall, POLYSORB® ID 37 administered at the doses of 0.5, 1, and 2 g of DEI/kg bw did not cause any effects on the embryo-foetal development in the rat.

On D6 and on D19 prior to dosing and then at 2 and 4 hours after dosing the plasma isosorbide analysis showed that the females were exposed to POLYSORB® ID 37. A dose related exposure was observed at 2 and 4 hours after dosing with a maximum at 2 hours. The plasma concentration before dosing was always lower than the LOQ.

### 3.6. Genotoxic Potential

#### 3.6.1. Ames Bacterial Reverse Mutation Assay

No evidence of toxicity was noted in the initial range-finding study. In strain TA102, in the presence and the absence of metabolic activation, and strain TA1535, in the absence of metabolic activation, the highest concentration of 5000 *μ*g/plate induced a decrease in the number of revertants. For these 2 strains the maximum dose was limited to 3000 *μ*g/plate.

There was no demonstrated mutagenicity with POLYSORB® ID 37 (see Table 2). The mean numbers of revertant colonies on negative control plates were all considered within acceptable levels, and the number of revertants was significantly elevated by the positive control treatments.

The determination of POLYSORB® ID 37 concentrations in treatment solutions was performed using a validated analytical method. The dosing results are thus considered as reliable. The results obtained for the concentration assay of POLYSORB® ID 37 in treatment solutions used in the Ames test were within the range 80–120% of the target values.

#### 3.6.2. Mouse Lymphoma TK Gene Mutation Assay

**Figure 2:**
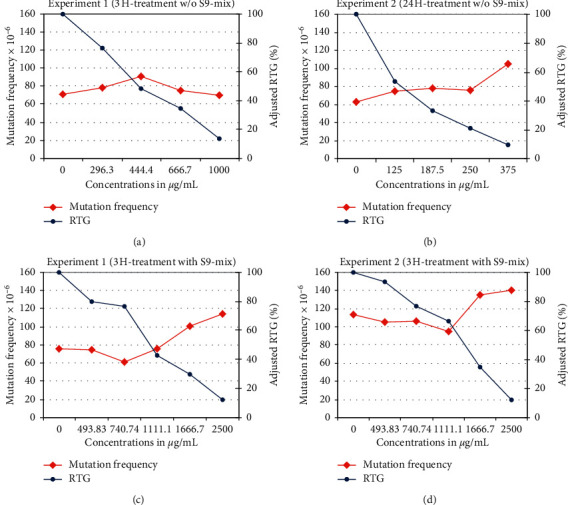
Results for the mouse lymphoma thymidine kinase gene mutation assay in absence and in presence of metabolic activation.

This study was performed to assess the potential of POLYSORB® ID 37 to induce mutations at the mouse lymphoma TK locus.  Figure 2 illustrates the data.

In Experiment 1, with a 3-hour treatment, with and without metabolic activation, the relative total growths of the L5178Y cells after treatment with the highest concentrations were 12.2% and 13.9%, at 2500 *μ*g/mL and 1000 *μ*g/mL, respectively. This was compared to positive control values of 63.8% (CPA with metabolic activation) and 69.8% (MMS: without metabolic activation). The induced mutation frequency for the highest dose group without metabolic activation was 20.6 mutants ×10^−6^ cells at the intermediary dose of 444.4 *μ*g/mL. The induced mutation frequency for the positive control was 300.3 mutants ×10^−6^ cells. In the group with metabolic activation the induced mutation frequency was 39 mutants ×10^−6^ cells at the highest dose of 2500 *μ*g/mL. The induced mutation frequency for the positive control was 373.6 mutants ×10^−6^ cells.

In Experiment 2, with 3-hour treatment with metabolic activation and 24 hours without metabolic activation, the relative total growths of the L5178Y cells after treatment with the highest concentration tested were 12.0% and 9.9%, for cells with and without metabolic activation at 2500 *μ*g/mL and 375 *μ*g/mL, respectively. This was compared to positive control values of 87.4% (CPA with metabolic activation) and 69.5% (MMS: without metabolic activation). Without metabolic activation, the highest induced mutation frequency was noted at the highest concentration of 375 *μ*g/mL, with 41.3 mutants ×10^−6^ cells. The induced mutation frequency for the positive control was 361 mutants ×10^−6^ cells. The induced mutation frequency was 26.8 mutants ×10^−6^ cells at the highest concentration of 2500 *μ*g/mL with metabolic activation compared to 306.3 mutants ×10^−6^ cells for the positive control. The spontaneous levels of mutants observed in the negative control and the response to known mutagen were in agreement with international recommendations [44].

In both experiments, there was no observed biological increase of mutants with and without metabolic activation.

Under the experimental conditions reported, POLYSORB® ID 37 is not considered to be mutagenic in the mouse lymphoma TK locus assay using the L5178Y cell line.

#### 3.6.3. Mammalian Erythrocyte Bone Marrow Micronucleus Test

An increase in the frequency of micronuclei was noted in the group treated with cyclophosphamide demonstrating the sensitivity of this animal strain as a clastogenic agent (Table 3). Neither mortality nor clinical signs were noted in animals treated with 500, 1000, or 2000 mg/kg bw of POLYSORB® ID 37 twice daily.

The ratio of polychromatic erythrocytes (PCE) to normochromatic erythrocytes (NCE) was established at each dose level. There was no decrease in the ratio PCE/NCE in the treatment groups compared to the negative control group, in treated males and females groups when analyzed separately or with sexes pooled.

Treatment with POLYSORB® ID 37, at all doses, had no effect on the frequency of micronucleated polychromatic erythrocytes compared to the control, in either the males or females groups or when the groups were combined. There was a decrease in the frequencies of micronucleated cells with 500 mg/kg bw twice daily in the male group and when both sexes were combined. Nevertheless, this decrease has no meaning in terms of genotoxic hazard.

Isosorbide concentrations in plasma after 30 minutes, 2 hours, and 4 hours of exposure in the male and female groups: mean isosorbide levels for male and female rats after 30 minutes reached 93.291, 106.946, and 225.744 *μ*g/mL in animals treated with 500, 1000, or 2000 mg/kg of POLYSORB® ID 37, respectively. After 2 hours of exposure, the mean isosorbide levels in male and female rats were 116.248, 191.342, and 427.627 *μ*g/mL in animals treated with 500, 1000, or 2000 mg/kg bw POLYSORB® ID 37, respectively. At 4 hours of exposure, the mean isosorbide levels for male and female rats reached 58.659, 112.553, and 287.321 *μ*g/mL in animals treated with 500, 1000, or 2000 mg/kg bw of POLYSORB® ID 37. A dose-response relationship was observed at each exposure time point, while a bell-curve was also observed for each dose in relation to the exposure time. Increased plasma isosorbide levels after oral administration of POLYSORB® ID 37 confirmed exposure of the rat bone marrow.

### 3.7. Ready Biodegradability CO_2_ Evolution (Modified Sturm Test)

The average percentage of biodegradability, after 28 days of incubation, was 83% and the pass level: 60% of ThCO_2_, was reached in a 10 d window, demonstrating the ready biodegradability of POLYSORB® ID 37.

## 4. Discussion

The toxicokinetic (TK) analysis showed that all animals treated by oral route were exposed to POLYSORB® ID 37, with males slightly more exposed than females. The short *C*_max_ demonstrated a rapid absorption by oral route. The short plasma half-life and the rapid urinary excretion suggest a relatively weak accumulation potential. By comparison, delayed excretion of DEHP was observed, in particular in adipose tissue [45]. POLYSORB® ID 37 presents a weak acute toxicity by oral and intravenous routes in mice and rats.

In repeated toxicity studies, mortalities observed after dosing with POLYSORB® ID 37 or C8/C10 were not directly attributed to a toxicological effect of POLYSORB® ID 37 or C8/C10 but to procedural difficulties encountered with the administration of the test article; said difficulties attributed the physical properties of the test compounds.

According to histopathology analysis, C8/C10 induced respiratory and gastric changes, which did decrease after a 14-day recovery period. Most of the clinical signs observed in C8/C10 groups were linked with the difficulties encountered during the treatment. The clinical signs observed in POLYSORB® ID 37 dose groups were not toxicologically relevant and were therefore not related to the treatment.

All the changes observed in clinical chemistry parameters were of low amplitude without dose dependency and physiological range and limits. Consequently, they were not considered as biologically relevant in POLYSORB® ID 37 dose groups. The increase in triglycerides observed in the C8/C10 and POLYSORB® ID 37 groups may have been due to the fat content of the two test products.

Histopathology showed that the incidence and severity of the gastric lesions observed in the C8/C10 group were more marked than in the POLYSORB® ID 37 groups, and they remained after dosing with C8/C10 whereas the lesions were totally reversed in POLYSORB® ID 37 dose groups.

Treatment with 2 g DEI/KG bw POLYSORB® ID 37 for 28 days led to a slight keratinized gastric hyperplasia which was completely reversed after a 14-day withdrawal, while 1 g DEI/kg bw POLYSORB® ID 37 for 28 days resulted in a slight keratinized gastric hyperplasia in only one animal which was considered as having little significance. Conversely, treatment with 1.57 mL/kg bw C8/C10 resulted in more severe keratinized gastric hyperplasia, ulceration of gastric and respiratory epithelia, and sometimes death. These gastric changes remained at a diminished degree after a 14-day period of recovery. The gastric effect is then attributed to the fatty acids that are locally irritating. There was no effect on endocrine function or the testis. It should be noted that at 2 g DEI/kg bw it did lead to a small decrease in the number and motility of sperm. The male reproductive tract is known to be particularly sensitive to phthalate exposure as treatment of adult male rats at high doses of certain phthalates (e.g., 2 g DEHP/kg bw/day) results in rapid and severe changes in the testis [46].

In the rodent liver DEHP activates the nuclear receptor peroxisome proliferator-activated receptor (PPAR) *α*. The peroxisome proliferator-induced increase in the number and size of peroxisomes in hepatocytes, so called “peroxisome proliferation” that results in elevation of fatty acid metabolism, is a hallmark response to these compounds in the liver. A link between peroxisome proliferation and tumor formation in rodents has been a predominant, albeit questioned, theory to explain the cause of a hepatocarcinogenic effect of peroxisome proliferators [47]. In the case of POLYSORB® ID 37, no sign of peroxisomes proliferation was noted in the rat after 4 and 13 weeks of treatment, on organ weight and at histology examination.

POLYSORB® ID 37 was devoid of genotoxic potential in a battery of both *in vitro* and *in vivo* tests. Previous studies on phthalate derivatives have yielded equivocal results. Tomita et al. [48] found that MEHP [mono-(2-ethylhexyl)-phthalate] but not DEHP [di-(2-ethylhexyl)-phthalate] exerted a dose-dependent DNA damaging effect to *B. subtilis* in rec-assay, that DEHP and MEHP showed mutagenic activities to *S. typhimurium* TA-100, with and without S-9 mix, respectively, and that MEHP produced not only the mutation in *E. coli* WP2B/*r* but also sister chromatid exchanges (SCE) in Chinese hamster V79 cells. It also induced 8AG/6TG-resistant gene mutations and chromosomal aberrations in the V79 cells. These authors demonstrated that both DEHP and MEHP induced 8AG/6TG-resistant mutation, chromosomal aberrations, and morphological transformation in the embryonic cells of the Syrian golden hamster.

Anderson et al. [49] reported that MEHP, the main metabolite of DEHP, was found to cause chromosome damage in CHO cells but was without effect in the sister chromatid exchange assay and hypoxanthine guanine phosphoribosyl transferase assay. These authors reported that DEHP was found to be a weak direct acting mutagen in *Salmonella typhimurium* strain TA100, the mutagenic activity of which could be abolished by rat liver microsomes (S9 mix). However Zeiger et al. [50] found a negative result in *Salmonella typhimurium* strains TA98, TA100, TA1535, and TA1537 without metabolic activation and in the presence of both rat and hamster liver S-9 metabolic activation systems. Agarwal et al. [51], Kirby et al. [52], and Yoshikawa et al. [53] demonstrated that DEHP and MEHP exhibited no mutagenicity in any of the strains of *Salmonella typhimurium* or in *Escherichia coli*. Kirby et al. [52] showed no genotoxic potential in the mouse lymphoma assay at the TK locus.

Anderson et al. [49] suggested that the clastogenicity and weak mutagenicity could present a possible contributory role for these compounds in the observed hepatocarcinogenicity of the phthalates. Finally, they showed that these compounds could produce DNA damage in human blood cells in the comet assay and also that rat liver microsomes could abolish the effect of DEHP. Thus, in the intact animal, no response may be observed.

The subcutaneous administration of 1–10 mg of DEHP to adult male mice on days 1, 5, and 10 followed by mating with untreated adult virgin females suggested a dominant lethal mutation effect in the treated mice. These effects tend to be more pronounced on the postmitotic stage of germ-cell development [54]. These results confirmed those of Autian [55] in mice treated for ten weeks at 1.37 ml/kg bw, by subcutaneous route.

In utero, some phthalates alter male and female reproductive tract differentiation. The mode of action in the male involves altered Leydig cell migration and differentiation and abnormal gonocytes development [56–59]. Leydig cell alterations result in reductions in foetal testis testosterone production and mRNA levels for key proteins in the steroidogenic pathway including StAR and CYP11, as well as insl-3, which is critical for gubernacular development and testis descent [60, 61]. Phthalates show little or limited estrogenic activity, and there is a building consensus that phthalates are indirect antiandrogenic agents. Phthalates and their monophthalate metabolites do not bind to the androgen receptor (AR) *in vitro* at concentrations of up to 10 *μ*M. In fact, phthalate toxicity toward Leydig cells depends on the dosage and time of exposure during development.

In contrast, POLYSORB® ID 37 did not induce any effects on the embryo-foetal development in the rat up to 2000 mg/kg bw/day and particularly no reproductive tract and gonads malformations were observed in the pups exposed in utero. The embryo-foetal and maternal NOAEL in the rat is 2000 mg/kg bw/day by oral route.

## 5. Conclusion

The main toxicological concerns for phthalates are liver toxicology, specifically the peroxisome proliferators' properties involved in rodent carcinogenesis and the hormone disruptor properties involved in embryo-foetal toxicity. It should be noted that equivocal genotoxic potential could be involved in liver carcinogenicity in rodents. In the present toxicity study, POLYSORB® ID 37 did not show any effect on liver toxicology or embryo-foetal toxicity. Given these findings, when compared to other phthalate substitutes, POLYSORB® ID 37 appears to be a safer compound. All of the toxicological and biodegradability studies were performed according to OECD Good Laboratory Practices and have affirmed it to be readily biodegradable and nontoxic to mammalian life, in the experimental conditions employed. Complementary studies should be performed including carcinogenicity studies, embryo-foetal studies, in a second nonrodent species, and two generation reproduction studies.

## Figures and Tables

**Table 1:** Characteristics and properties of POLYSORB® ID 37.

Chemical source	
Name	DEI
Formula	Not applicable since UVCB
Chemical name	Isosorbide diesters Fatty acids, C8-10, diesters with 1,4 : 3,6-dianhydro-D-glucitol
CAS#	1215036-04-6
List number	700-073-5
Molecular formula	As it is an UVCB, we cannot provide the exact molecular formula
Molecular weight	Ca. 410 g
Melting point	Between −5°C and +13°C (freezing point)
Boiling point	Between 355.9°C and 376.6°C at ca. 97 kPa
Density	1.02–1.03 at 20°C
Vapour pressure	0.03 Pa at 25°C
Partition coefficient (log Kow)	>2.9 at 70°C (pH 4.48)
Water solubility	≤5 mg/L at 20°C (pH 7)
Surface tension	Ca. 50.8 mN/m at 20°C (4 mg/L)
Flash point	201.0 ± 0.5°C [KS, K1]
Explosiveness	No explosive properties
Oxidizing properties	No oxidizing properties
Dynamic viscosity	67.6–73.4 mPa s at 20°C 22.9 and 24.5 mPa s at 40°C

**Table 2:** Results for the Ames bacterial reverse mutation assay with and without metabolic activation, 2 independent assays.

TA 1535			TA 1537		TA 98		TA 100		TA 102	
Doses (*μ*/plate)	Nb revertants/plate	Induction ratio (a)	Nb revertants/plate	Induction ratio (a)	Nb revertants/plate	Induction ratio (a)	Nb revertants/plate	Induction ratio (a)	Nb revertants/plate	Induction ratio (a)
Without metabolic activation										
Assay 1										
(b)	1338.7	97.7	655.3	131.1	523.3	43.6	1320.0	8.4	1645.3	8.2
0	18.8	—	4.8	—	14.7	—	150.7	—	168.5	—
50	15.3	0.8	4.7	1.0	14.7	1.0	143.0	0.9	173.7	1.0
150	12.3	0.7	5.7	1.2	10.0	0.7	148.3	1.0	197.7	1.2
500	13.0	0.7	5.7	1.2	7.0	0.5	142.7	0.9	165.3	1.0
1500	13.0	0.7	5.7	1.2	6.3	0.4	100.7	0.7	132.3	0.8
3000	10.7	0.6							89.7	0.5
5000			4.3	0.9	5.3	0.4	36.3	0.2		
Assay 2										
(b)	427.3	67.8	466.0	141.2	550.0	41.4	491.0	5.1	753.3	5.0
0	7.3	—	3.3	—	14.3	—	9507	—	134.2	—
50	6.3	0.9	3.0	0.9	11.0	0.8	90.3	0.9	132.7	1.0
150	5.7	0.8	5.3	1.6	10.7	0.7	85.7	0.9	132.7	1.0
500	5.0	0.7	2.0	0.6	12.7	0.9	81.0	0.8	133.7	1.0
1500	3.3	0.5	1.3	0.4	8.3	0.6	90.7	0.9	115.3	0.9
3000	4.3	0.6							91.7	0.7
5000			0.7	0.2	6.7	0.5	63.0	0.7		

With metabolic activation										
Assay 1										
(c)	327.3	44.8	228.3	61.7	1494.7	78.7	1625.3	153	801.3	5.0
0	7.0	—	5.0	—	19.3	—	111.3	—	235.3	—
50	11.7	1.7	10.3	2.1	16.7	0.9	141.3	1.3	164.0	0.7
150	9.3	1.3	5.3	1.1	17.0	0.9	154.0	1.4	187.3	0.8
500	11.3	1.6	5.7	1.1	24.3	1.3	155.0	1.4	163.3	0.7
1500	6.7	1.0	8.0	1.6	22.3	1.2	139.7	1.3	139.0	0.6
3000									49.3	0.2
5000	8.3	1.2	3.0	0.6	21.7	1.1	140.7	1.3		
Assay 2										
(c)	242.7	20.2	220.0	34.9	2405.3	80.2	1680.0	15.9	791.3	2.7
0	10.2	—	6.8	—	33.3	—	103.0	—	280.3	—
50	11.3	1.1	7.7	1.1	23.3	0.7	99.3	1.0	183.7	0.7
150	9.0	0.9	9.0	1.3	30.0	0.9	82.7	0.8	260.7	0.9
500	8.3	0.8	7.3	1.1	23.7	0.7	91.3	0.9	176.3	0.6
1500	11.3	1.1	4.3	0.6	18.3	0.5	87.3	0.8	162.3	0.6
3000									64.3	0.2
5000	4.7	0.5	0.7	0.8	19.0	0.6	66.0	0.6		

The second assay in presence of metabolic activation was carried out following the preincubation method. (a) Induction Ratio = number of revertants in the treated/number of revertants in the controlReference positive compounds (*μ*g/plate). (b) TA1535 and TA100: Sodium azide 1; TA1537: 9-amino-acridine 50; TA98: 2-nitrofluorene 2; TA102: Mitomycin C 0.125. (c ) TA1535, TA1537, TA98, TA100: 2-anthramine 2 (without pre-incubation), 1 (with pre-incubation); TA102: benzo(a)pyrene 2.

**Table 3:** Results for the *in vivo* micronucleus test in the OFA Sprague-Dawley male and female rats.

Doses in mg/kg/day (X2)		PCE/NCE ratio (mean ± SD)	MNC for 1000 PCE (mean ± SD)
0	M	1.38 ± 0.46	0.70 ± 0.27
	F	1.01 ± 0.27	0.40 ± 0.42
	*M* + *F*	1.20 ± 0.41	0.55 ± 0.37

CPA 25 mg/kg/day (×1)	M	0.72^*∗*^ ± 0.05	7.60^*∗∗*^ ± 1.64
	F	0.60^*∗*^ ± 0.06	6.60^*∗∗*^ ± 1.08
	*M* + *F*	0.66^*∗∗∗*^ ± 0.08	7.10^*∗∗∗*^ ± 1.41

2000	M	1.04 ± 0.22	0.80 ± 0.27
	F	1.21 ± 0.44	0.90 ± 0.55
	*M* + *F*	1.12 ± 0.34	0.85 ± 0.41

1000	M	0.93 ± 0.23	0.60 ± 0.65
	F	0.94 ± 0.11	0.30 ± 0.27
	*M* + *F*	0.93 ± 0.17	0.45 ± 0.50

500	M	1.06 ± 0.24	0.20^*∗*^ ± 0.27
	F	0.89 ± 0.28	0.20 ± 0.27
	*M* + *F*	0.97 ± 0.24	0.20^*∗*^ + 0.26

^*∗*^Statistically significant at the threshold of *p* < 0.05, ^*∗∗*^statistically significant at the threshold of *p* < 0.01, ^*∗∗∗*^statistically significant at the threshold of *p* < 0.001 (PCE/NCE ratio: Student's *t*-test; micronucleates frequency: Mann‐Whitney U test). PCE: polychromatic erythrocytes; NCE: normochromatic erythrocytes; MNC: micronucleates; M: male rats; F: female rats.

## Data Availability

Data are archived following GLP Regulations at either Institut Pasteur de Lille, ERBC, or INERIS. They are however available on request to Mrs. Aouatif Chentouf.

## Additional Points

The toxicology studies given in Section 1.1 were started before 2009 as part of a REACH registration dossier. The product that is currently on the market is under the name of POLYSORB® ID46 in ROQUETTE.
